# A Simple Model for Inducing Optimal Increase of* SDF-1* with Aminoglycoside Ototoxicity

**DOI:** 10.1155/2017/4630241

**Published:** 2017-12-21

**Authors:** Hyun Mi Ju, Sun Hee Lee, Jin Sil Choi, Young Joon Seo

**Affiliations:** ^1^Laboratory of Smile Snail, Yonsei University Wonju College of Medicine, Wonju, Republic of Korea; ^2^Department of Otorhinolaryngology, Yonsei University Wonju College of Medicine, Wonju, Republic of Korea

## Abstract

**Objectives:**

As a homing factor of stem cell, stromal derived factor-1* (SDF-1)* is important for the regenerative research in ototoxicity. Mice models with aminoglycoside ototoxicity have been widely used to study the regeneration capacity of MSCs in repair of cochlear injury. We developed a mouse model with maximal increase in* SDF-1* levels in the inner ear, according to the “one-shot” doses of kanamycin and furosemide.

**Methods:**

C57BL/6 mice had kanamycin (420, 550, and 600 mg/kg) dissolved in PBS, followed by an intraperitoneal injection of furosemide (130 mg/kg). The injuries of inner ear were measured with hearing thresholds, histology, and outer hair cell counts at 0, 3, 5, 7, 10, and 14 days before the sacrifice. The levels of* SDF-1* in the inner ear were tested by real-time RT-PCR and immunohistochemistry.

**Results:**

There were a significant reduction in hearing thresholds and a maximal increase of* SDF-1* levels in the furosemide 130 mg/kg + kanamycin 550 mg/kg group, but severe hearing deterioration over time was observed in the furosemide 130 mg/kg + kanamycin 600 mg/kg group and four mice were dead.* SDF-1* was detected mostly in the stria vascularis and organ of Corti showing the highest increase in expression.

**Conclusion:**

We observed optimal induction of the stem cell homing factor in the newly generated aminoglycoside-induced ototoxicity mouse model using a “one-shot” protocol. This study regarding high* SDF-1* levels in our mouse model of ototoxicity would play a major role in the development of therapeutic agents using MSC homing.

## 1. Introduction

Stromal derived factor-1* (SDF-1)* is a cytokine for stimulating the homing of stem cells into injured organs. The expression of* SDF-1* in injured tissue correlates with recruitment of stem cells and tissue regeneration. Recent studies have shown that homing of mesenchymal stem cells (MSCs) across the blood-brain barrier (BBB) occurred in ischemic brain tissue. Myocardial protection by homing of stem cells was also shown in myocardial infarction via mobilization of the stem cells into the injured myocardial tissue and increase in local angiogenesis after myocardial infarction.

Mice models with aminoglycoside ototoxicity have been widely used to study the regeneration capacity of MSCs in repair of cochlear injury. Several studies showed that mice can be used as models for aminoglycoside-induced hearing loss using a “one-shot” protocol, in which a single dose of kanamycin is accompanied by a dose of the loop diuretic furosemide [1, 2].

In the case of cochlear homing, Tan et al. [3] demonstrated that upregulation of* SDF-1* in the spiral ligament after acoustic deafening could promote the homing capability of bone marrow-derived cells to an injured cochlea. Another study showed that efficient invasion of MSCs to the inner tissue occurred when MSCs, which had enhanced expression of the* SDF-1* receptor and the C-X-C chemokine receptor type 4 (CXCR4), were transplanted in the lateral semicircular canal [4].

In the field of ear research, researches are being actively carried out to bring about the regeneration of hair cells using stem cell homing [4]. To prove that this ototoxicity mice model, in which* SDF-1* is markedly increased, could be the appropriate model to study homing, we confirmed the changes in* SDF-1* levels with increasing hearing thresholds based on the various conditions of one-shot ototoxicity. Thereafter, we demonstrated an effective homing phenomenon in a mouse model with maximal increase in* SDF-1* levels in the inner ear.

## 2. Method

### 2.1. Animals

87 male C57BL/6 mice, including 24 mice for the changes of hearing thresholds after the ototoxicity drugs, were allowed free access to water and regular mouse diet and were kept at room temperature under a standard 12 h light/dark cycle for 1 week of acclimatization before the experiments. The animals were 5 weeks old and weighed approximately 18–25 g. The mice were anaesthetized by intraperitoneal injection of 30 mg/kg tiletamine-zolazepam (Zoletil, 500 mg/vial; Virbac, Carros, France) and 10 mg/kg xylazine (Rompun; Bayer Korea, Ansan, Korea) and sacrificed by decapitation. The animals underwent cardiac perfusion with phosphate-buffered saline (PBS: Dulbecco's formula modified, ICN Biochemicals, England) before tissue harvest. The temporal bones were dissected, and the bony shells of the cochlea and vestibule were removed in chloride-free physiological saline. The animal experiments were conducted in accordance with the guidelines of the Institutional Animal Care and Use Committee of Yonsei University, Korea (YWC-150728-1, YWC-160826-1).

### 2.2. Ototoxic Drug Administration

**Figure 1:**
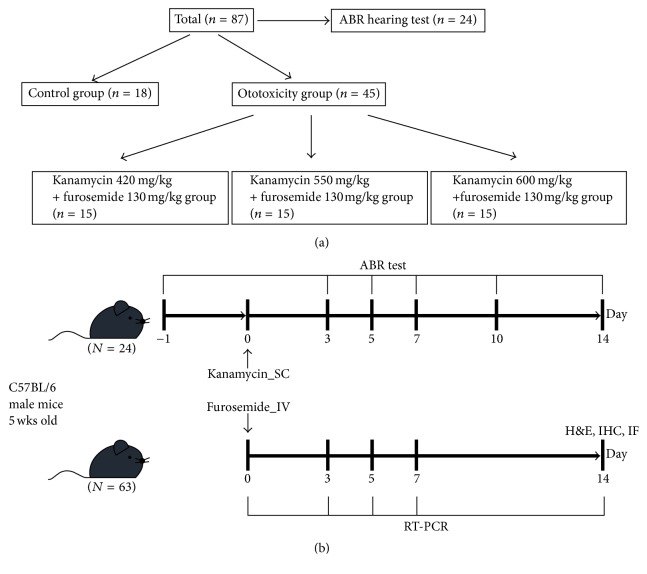
The study design for the mice model showing optimal increase of* SDF-1* with aminoglycoside ototoxicity. (a) Total population involving 63 male C57BL/6 mice (including 18 control mice). Mice were treated according to the following protocol: to record auditory brainstem responses (ABRs) 24 h after the second LPS injection, mice were injected with kanamycin (420, 550, and 600 mg/kg) and, 30 min later, with furosemide (130 mg/kg). (b) Auditory brainstem responses (ABRs) were tested on days 3, 5, 7, and 14 after kanamycin-furosemide administration. On the 14th day, fixative was perfused through the heart and cochleas were harvested for histologic analysis.

The first injection for each mouse was given at the beginning of the daily light cycle. The three subgroups (*n* = 15 for each group) in the ototoxicity group received subcutaneous injection of kanamycin (420, 550, and 600 mg/kg; Sigma-Aldrich Oakville, ON, Canada) dissolved in PBS, followed by an intraperitoneal injection of furosemide (130 mg/kg; Sigma-Aldrich, Oakville, ON, Canada) via the tail vein after 30 min [5]. The mice in the sham control group received a subcutaneous injection of saline, followed by another tail vein injection of saline 30 min later. Mice showing signs of severe dehydration or other significant illness were sacrificed. All animals were monitored by trained animal care technologists supervised by a veterinarian. All animals survived the drug administration (Figure 1).

### 2.3. Testing of Hearing Ability

Pretest auditory brainstem response (ABR) thresholds were measured in the 24 mice (6 mice/each group) 24 h prior to the first drug administration. Each animal was gently anaesthetized with an intraperitoneal injection of ketamine (100 mg/kg; Yuhan Corporation, Seoul, Korea) and xylazine (1 mg/kg; Rompun, Korea Bayer, Ansan, Korea) and kept warm using a heating pad. Subdermal needle electrodes were placed at the scalp vertex (inverting), posterior bulla (noninverting), and lower back (ground) for recording ABR in anaesthetized mice. The test stimuli were clicks generated using the BioSigRP (Tucker-Davis Technologies, Inc.). The stimulus intensity decreased gradually in 5-dB (decibel) steps until a visually discernible ABR waveform disappeared, and the lowest sound level that caused this waveform was defined as the “threshold.” Scanning time was 10 ms, and 1024 sweeps were averaged with 300–3000 Hz filtering band-width. Threshold shifts are reported as the difference between the pre- and posttest ABR thresholds. Posttest ABR thresholds after kanamycin and furosemide administration were measured at 3, 5, 7, 10, and 14 days without any scarification to observe the changes of hearing threshold after the ototoxicity drugs.

### 2.4. Histological Assessment and Immunohistochemistry

Normal and ototoxic drug-administrated mice were sacrificed after 14 days of treatment. The temporal bones were removed, and the apex of the cochlea, the round window, and the oval window were punctured. A fixative was perfused on right side cochleas of three animals in each group through the cochlear apex with 4% paraformaldehyde (Biosesang, Seongnam, Korea), and the sample was immersed in fixative for 24 h at 4°C. Cochleas were decalcified by immersion in Calci-Clear Rapid (National Diagnostics, Atlanta, GA, USA) for 24 h, dehydrated in 30% sucrose (Sigma-Aldrich, Gillingham, UK) for 24 h, embedded in the optimal cutting temperature (OCT) compound (Leica, Bensheim, Germany), and sectioned from 2 to 10 *μ*m thickness using a cryostat (Leica CM1850 Cryostat; Leica, Wetzlar, German). A standard hematoxylin and eosin (H & E) staining protocol was followed, with a 1–3 min incubation in hematoxylin and 30–60 s staining with eosin, before mounting the samples.

Immunohistochemistry for* SDF-1* was performed on the cryosections of the cochlea in each group. The slide samples were incubated with appropriate primary antibodies as follows. Antibody against* SDF-1* 1 (Abcam, Inc., Cat #ab18919) was used. Sections were incubated with the primary antibody overnight at 4°C. After washing thrice with 0.1 M PBS, the sections were incubated with an appropriate biotin-tagged secondary antibody at room temperature for 1 h. Thereafter, the sections were incubated in an avidin-biotin-peroxidase complex solution (Vector Laboratories, Inc., Burlingame, CA, USA) and developed with diaminobenzidine substrate kit (Vector Laboratories, Inc.) after washing thrice with 0.1 M PBS. Then, the sections were dehydrated, mounted, and visualized with a BX50 microscope (Olympus, Tokyo, Japan), and digital images were captured.

### 2.5. Immunofluorescent Microscopy

The temporal bones of the left side in three mice of each group that were sacrificed for the histology were fixed and decalcified as described above. Subsequently, the basilar membrane was dissected under a dissecting microscope, and the stria vascularis and the tectorial membrane were removed. To identify F-actin and cell nucleus in the sensory epithelium, phalloidin-FITC (Sigma-Aldrich, Chemie BV, Zwijndrecht, the Netherlands) was applied for 40 min at room temperature in a dark room. The fluorescent signals were visualized using a BX50 microscope (Olympus, Tokyo, Japan) and digital images were captured. The outer hair cells in the sensory epithelium were counted from a minimum of 10 captures per cochlea, including the apical, mid, and basal turns.

### 2.6. Real-Time Reverse Transcription-Polymerase Chain Reaction (RT-PCR) Analysis

Total RNA was isolated with the TRIzol Reagent (Invitrogen, Carlsbad, CA) from the sensory epithelium of right cochlea of three mice in each group, which were sacrificed 3, 5, 7, and 14 days after the treatment. On day 0 before the ototoxicity, the results were replaced by those from the control group (*n* = 6). Total RNA was subjected to reverse transcription using the SYBR® Select master mix (Applied Biosystems, CA, USA) following the manufacturer's protocol. Real-time RT-PCR was performed using Applied Biosystems sequence detection system 7900 to quantify* SDF-1* levels. The following primers were used for sequencing:* SDF-1*, forward: 5′-CGC CAG AGC CAA CGT CAA GC-3′ and reverse: 5′-TTT GGG TCA ATG CAC ACT TG-3′; *β*-actin, forward: 5′-CGT GCG TGA CAT CCA AGA GAA-3′ and reverse: 5′-TGG ATG CCA CAG GAT TCC AT-3′. To exclude the possibility of genomic DNA amplification during PCR, no-template controls were performed and accepted when the Ct value was at least nine cycles greater than the template run. Measurements were performed in duplicate and accepted if the difference in Ct values between the duplicates was less than 1. The real-time PCR data were normalized to the level of *β*-actin, and the relative quantity of mRNA was determined using the comparative cycle threshold method.

### 2.7. Statistical Analysis

Statistical analysis was performed using the SPSS statistical package version 17.0 (SPSS, Chicago, IL, USA). Descriptive results of continuous variables are expressed as mean ± standard deviation (SD) for normal distribution variables. Means were compared by the 2-way analysis of variance (ANOVA) for ABR tests and Mann–Whitney test for counts of hair cells and* SDF-1*. The level of statistical significance was set at 0.05.

## 3. Results

### 3.1. Changes in the Hearing Thresholds of the Kanamycin/Furosemide-Induced Mouse Model of Ototoxicity

**Figure 2:**
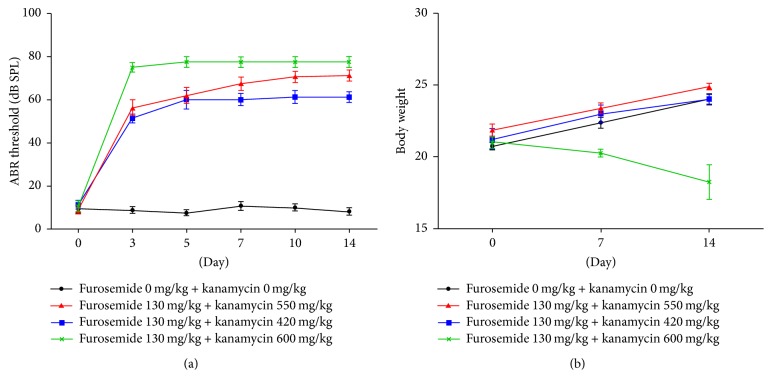
The shifts of auditory brainstem response (ABR) thresholds in the mouse model of ototoxicity were measured after injections with different doses of furosemide and kanamycin. In the groups receiving furosemide 130 mg/kg + kanamycin 420 mg/kg, furosemide 130 mg/kg + kanamycin 550 mg/kg, and furosemide 130 mg/kg + kanamycin 600 mg/kg, the ABR thresholds were significantly increased compared to that of the control group at all tested frequencies (*P* < 0.01). The data are expressed as the mean ± SD of 5 mice in each group and were analyzed using Student's *t*-test.

ABR evaluations were performed at 0, 3, 5, 7, 10, and 14 days after drug administration to test whether the combined effect of kanamycin/furosemide is able to induce hearing loss in mice. We compared the average ABR thresholds for the four groups, which was measured with click sounds (Figure 2(a)). The ABR thresholds were significantly elevated in the groups receiving both drugs, namely, furosemide 130 mg/kg + kanamycin 420 mg/kg, furosemide 130 mg/kg + kanamycin 550 mg/kg, and furosemide 130 mg/kg + kanamycin 600 mg/kg, compared to that of the control group at each frequency (*P* < 0.01). Abrupt changes in threshold in the ototoxicity groups occurred after 3 days of drug administration. High doses of kanamycin caused more severe hearing impairment in the third to the fourteenth day posttreatment, as seen by an increase in the ABR threshold in the furosemide 130 mg/kg + kanamycin 600 mg/kg group than in the furosemide 130 mg/kg + kanamycin 420 and 550 mg/kg groups. Final hearing levels worsened with administration of different concentrations of kanamycin; however, there were no significant differences among them. Despite definite changes in the hearing threshold, the body weights of mice treated with kanamycin 600 mg/kg decreased, and 4 among 15 mice died of urinary insufficiency within 7 days (Figure 2(b)). However, no such problem occurred in mice treated with the other doses of kanamycin.

### 3.2. Cochlear Histopathology of Kanamycin/Furosemide-Treated Mice

**Figure 3:**
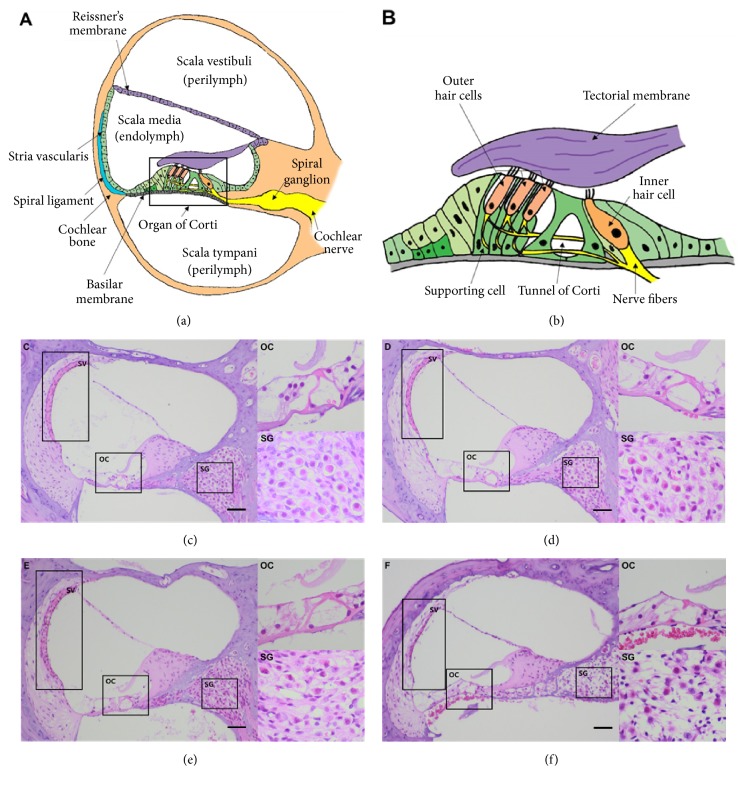
The cochlear morphology in the mouse model of ototoxicity analyzed after furosemide and kanamycin treatments showed cochlear degeneration. (a) and (b) show the normal microstructures of cochlea, especially of the organ of Corti and the spinal ganglion. We compared the hematoxylin and eosin staining in the cryosections from (c) normal mice and mice treated with furosemide, (d) 130 mg/kg + kanamycin 420 mg/kg, (e) furosemide 130 mg/kg + kanamycin 550 mg/kg, and (f) furosemide 130 mg/kg + kanamycin 600 mg/kg. SV: scala vestibuli; SM: scala media; ST: scala tympani; RM: Reissner's membrane; TM: tectorial membrane; BM: basilar membrane; SG: spiral ganglion; OHC: outer hair cells; IHC: inner hair cells. Scale bars: 20 *μ*m.

To examine the ototoxic effects of furosemide and kanamycin, structural changes in the inner ear were examined by H & E staining of cochlear sections 14 days after the drug administration. We observed a loss of inner and outer hair cells in mice treated with a combination of kanamycin and furosemide, which caused damage of the organ of Corti (Figure 3). The loss of outer hair cells in the organ of Corti was higher in the cochlea exposed to kanamycin/furosemide than in the normal cochlea. At the same time, there was a reduction in the number of spiral ganglia cells and thinning of the stria vascularis in the groups receiving both the drugs. High doses of kanamycin (550 and 600 mg/kg) caused more severe structural changes.

### 3.3. Hair Cell Counts in Kanamycin/Furosemide-Treated Mice

**Figure 4:**
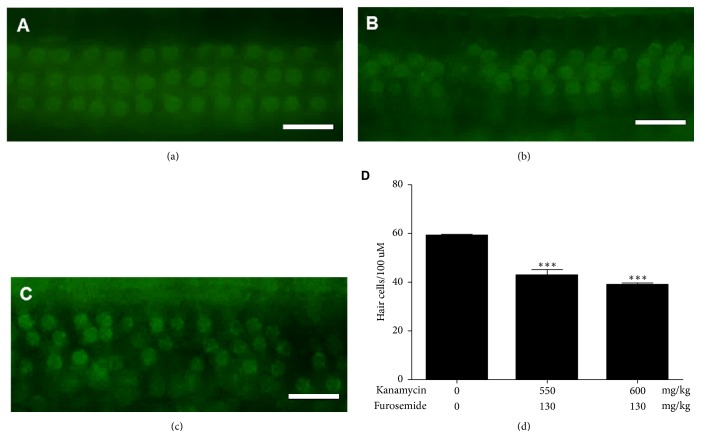
Phalloidin-FITC immunofluorescence shows loss of outer hair cells. Immunofluorescence in sections from mice treated with (a) furosemide 130 mg/kg + kanamycin 420 mg/kg, (b) furosemide 130 mg/kg + kanamycin 550 mg/kg, and (c) furosemide 130 mg/kg + kanamycin 600 mg/kg. The outer hair cells in the three layers show dysmorphic, out of line cells and deletion of cells. (d) There were significant differences for counts of outer hair cells in the kanamycin 550 mg/kg and 600 mg/kg treated groups than in the sham control group.

The process of degeneration in the organ of Corti 14 days after drug administration was examined by the structure analysis using immunofluorescence microscopy (Figure 4). Cochleas from furosemide 130 mg/kg + kanamycin 550 mg/kg and furosemide 130 mg/kg + kanamycin 600 mg/kg treated mice were analyzed. The alignment of outer hair cells (OHCs), indicated by their V-shaped bundles, indicated that the organization of the organ of Corti and the stereocilia bundle integrity were well-maintained at this stage. In contrast, damage to the stereocilia was observed in the furosemide 130 mg/kg + kanamycin 550 mg/kg treated mice, which was more evident in mice treated with furosemide 130 mg/kg + kanamycin 600 mg/kg, particularly at the top of their V-shaped bundles. In addition, the hair cells presented an increasingly disorderly rearrangement, and some OHC stereocilia and the cuticular plate were absent. To quantify bundle damage caused by furosemide and kanamycin sulfate, the total number of bundles of the three-layered outer hair cells was counted. Combined injection of furosemide and kanamycin sulfate caused a significant reduction in the number of bundles of the inner and outer hair cells in the furosemide 130 mg/kg + kanamycin 550 mg/kg and furosemide 130 mg/kg + kanamycin 600 mg/kg groups compared to that of the control group (*P* < 0.01).

### 3.4. Changes in* SDF-1* Level in the Kanamycin/Furosemide-Treated Mice

**Figure 5:**
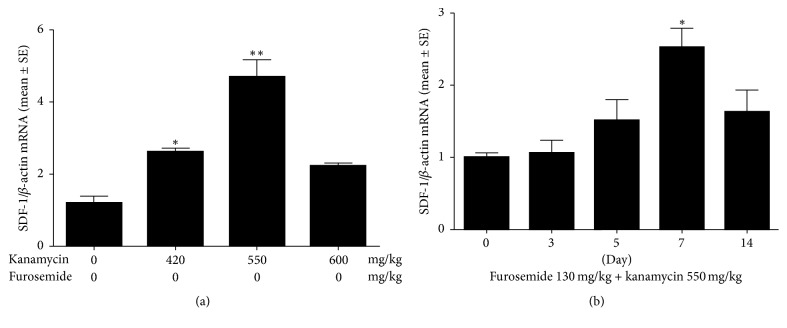
The changes in* SDF-1* mRNA levels in the sensory epithelium of inner ear after dose and time-dependent treatment with furosemide and kanamycin were assessed by real-time RT-PCR. (a) The maximal increase in* SDF-1* occurred at the dose of furosemide 130 mg/kg + kanamycin 550 mg/kg. (b)* SDF-1* levels increased significantly (more than 2.5-fold) on the 7th day after treatment with furosemide 130 mg/kg + kanamycin 550 compared with that of the control (*P* < 0.01).

**Figure 6:**
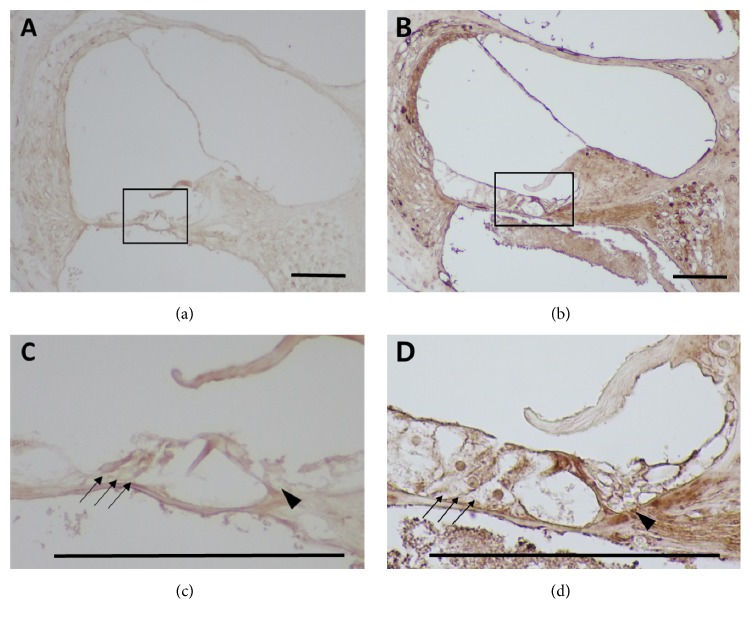
The distribution of* SDF-1* in the cochlea after treatment with furosemide 130 mg/kg + kanamycin 550 mg/kg was assessed by immunohistochemistry.* SDF-1* was detected in the stria vascularis, Reissner's membrane, organ of Corti, and spinal ganglion. (a) ×200 magnified image of the cochlea of normal control mice. (b) ×200 magnified image of the cochlea of the treated mice with furosemide 130 mg/kg + kanamycin 550 mg/kg. Furthermore, the organ of Corti showed the highest increase (d) in expression, compared to the control (c). Both outer and inner hair cells showed increased levels of* SDF-1*. Scale bars: 50 *μ*m.

*SDF-1* is an important chemokine required for the homing of mesenchyme-derived stem cells. We observed that the mRNA levels of* SDF-1* increased with concentration of kanamycin (Figure 5), reaching maxima in the furosemide 130 mg/kg + kanamycin 550 mg/kg group. However, the levels decreased in the furosemide 130 mg/kg + kanamycin 600 mg/kg group. Next, we measured the change in* SDF-1* with time using 550 mg/kg kanamycin. The hearing thresholds dropped suddenly on the third day. In contrast, the mRNA levels of* SDF-1* were highest on the 7th day and decreased afterwards. Next, we investigated the extent and timing of the increase in the protein levels of* SDF-1* (Figure 6).* SDF-1* was detected in the stria vascularis, Reissner's membrane, organ of Corti, and the spinal ganglion, confirming that* SDF-1* was expressed mostly in the damaged area as ototoxicity progressed in the second week. Furthermore, the stria vascularis and organ of Corti showed the highest increase in expression. Both outer and inner hair cells also showed increased expression.

## 4. Discussion

Stem cell homing via circulation to the bone marrow is the first critical step in regenerative medicine [6, 7]. Stem cell homing is one of the crucial mechanisms that has to be activated for efficient cell delivery to the inner ear [4]. Sensory hair cells of the inner ear are responsible for conducting auditory stimulation. The hair cells in the inner ear are never replaced and are regenerated after injury. Injection of neural stem cells for homing to the injured cochlea is required to induce regeneration via the supporting cells in the organ of Corti following inner ear trauma [8]. However, there are only few animal models for an increasing number of homing factors, showing an obvious dearth of appropriate study models. Tan et al. [3] studied the homing capability of bone marrow-derived stem cells to the deafened cochlea in the rat model 3 months after acoustic deafening. Here, we attempted to develop a mouse model with an optimal increase of* SDF-1* for homing studies. This would be a useful tool to evaluate the efficacy of stem cell therapy for other related researchers.

*SDF-1 (CXCL12)*, a member of the C-X-C family of chemokines, plays an important role in cell migration at the injured site [9]. CXCR4 on the circulating stem cells are trapped by* SDF-1*, which represent high-affinity cell surface integrins. In the presence of the* SDF-1-CXCR4* combination, the cells stop rolling, disseminate, and migrate through the vascular endothelium towards the chemokine gradient [10, 11]. Kamiya [4] transplanted MSCs in the cochlea after the induction of* SDF-1* in* CX26*-deficient mice, which was used as a model of hereditary hearing loss [12]. We observed optimal induction of the stem cell homing factor in the newly generated aminoglycoside-induced ototoxicity mouse model using a “one-shot” protocol. There was a significant reduction in hearing thresholds and a maximal increase of* SDF-1* levels when furosemide 130 mg/kg + kanamycin 550 mg/kg was used to treat the mice. Severe ototoxicity was observed in the furosemide 130 mg/kg + kanamycin 600 mg/kg group, which was accompanied by distorted structures in the organ of Corti, cell death, and weight loss among the population with urinary insufficiency. Since high doses of aminoglycoside cause drug-induced nephrotoxicity [13, 14], the nephrotoxicity induced by kanamycin 600 mg/kg, which manifested as a reduction in urine volume, was considered as a case of renal failure. There were no such symptoms in the other groups, which were treated with doses of kanamycin less than 550 mg/kg. The hearing thresholds decreased abruptly on the 3rd day and plateaued on the 5th day after the ototoxicity injury. We consider the 7th day posttreatment as the optimal day for the homing mouse model with aminoglycoside ototoxicity, because* SDF-1* levels peaked on the 7th day after the drug treatment.

Immunohistochemistry performed on the 7th day posttreatment showed that* SDF-1* was distributed diffusely in the stria vascularis, Reissner's membrane, organ of Corti, and spinal ganglion. This observation corroborates the results obtained with kanamycin-mediated injury. Extensive destruction of the outer hair cells in the triple-layer arrays and decreased count and vacuolization of the spiral ganglion cells were typical initial characteristics of cochlea exposed to ototoxicity [15]. However, dominant staining with the* SDF-1* antibody in the scala vascularis represented the possibility of increasing the homing effect of the stem cells, because scala vascularis consists of condensed vascular structures.

The “one-shot” injection with combinations of kanamycin and furosemide in the mouse model of ototoxicity is a novel technique for inducing local inner ear injury [16–18]. Abbas and Rivolta [1] showed the efficiency of the “one-shot” approach. A single dose regimen might be a reliable model for ototoxicity-mediated hearing loss with kanamycin and adequate for inducing rapid and profound hearing impairment. Furthermore, it reduces the risks associated with chronic treatment of aminoglycoside and is thus preferred for animal welfare. Although Abbas et al. [1] used 400–500 mg/kg kanamycin, followed by an intraperitoneal injection of furosemide 100 mg/kg after 20–30 mins in gerbils, we used various doses of kanamycin (420–600 mg/kg) with 130 mg/kg furosemide in C57BL/6 mice. We observed that ototoxicity-mediated hearing impairment occurred upon using kanamycin doses equal to or greater than 420 mg/kg. However, we conclude that the optimal dose of kanamycin for the homing mice model of ototoxicity is 550 mg/kg. In the future, we will use this animal model to study the MSC homing in the ototoxicity mice after the injection of MSCs intratympanically or intravenously, which have increased potency of* CXCR4* with hypoxic conditions.

Investigation regarding the mechanism of ototoxicity induced by the combination of kanamycin-furosemide is important for understanding stem cell homing in the inner ear [19–21]. In future, we would use the* SDF-1/CXCR4* axis to induce MSC homing in the injured cochlear site in this mice model through the administration of MSC with elevated* CXCR4* expression. Eventually, studies regarding high* SDF-1* levels in our mouse model of ototoxicity would play a major role in the development of therapeutic agents using MSC homing.
